# Hepatotoxicity and the role of the gut-liver axis in rats after oral administration of titanium dioxide nanoparticles

**DOI:** 10.1186/s12989-019-0332-2

**Published:** 2019-12-27

**Authors:** Zhangjian Chen, Di Zhou, Shuo Han, Shupei Zhou, Guang Jia

**Affiliations:** 1 Department of Occupational and Environmental Health Sciences, School of Public Health, Peking University, Beijing, 100191 China; 2 Beijing Key Laboratory of Toxicological Research and Risk Assessment for Food Safety, School of Public Health, Peking University, Beijing, 100191 China; 3 Department of Laboratory Animal Science, Health Science Center, Peking University, Beijing, 100191 China

**Keywords:** Gut microbiota, Hepatotoxicity, Nanomaterials, Titanium dioxide, Gut-liver axis

## Abstract

**Background:**

Due to its excellent physicochemical properties and wide applications in consumer goods, titanium dioxide nanoparticles (TiO_2_ NPs) have been increasingly exposed to the environment and the public. However, the health effects of oral exposure of TiO_2_ NPs are still controversial. This study aimed to illustrate the hepatotoxicity induced by TiO_2_ NPs and the underlying mechanisms. Rats were administered with TiO_2_ NPs (29 nm) orally at exposure doses of 0, 2, 10, 50 mg/kg daily for 90 days. Changes in the gut microbiota and hepatic metabolomics were analyzed to explore the role of the gut-liver axis in the hepatotoxicity induced by TiO_2_ NPs.

**Results:**

TiO_2_ NPs caused slight hepatotoxicity, including clear mitochondrial swelling, after subchronic oral exposure at 50 mg/kg. Liver metabolomics analysis showed that 29 metabolites and two metabolic pathways changed significantly in exposed rats. Glutamate, glutamine, and glutathione were the key metabolites leading the generation of energy-related metabolic disorders and imbalance of oxidation/antioxidation. 16S rDNA sequencing analysis showed that the diversity of gut microbiota in rats increased in a dose-dependent manner. The abundance of *Lactobacillus_reuteri* increased and the abundance of *Romboutsia* decreased significantly in feces of TiO_2_ NPs-exposed rats, leading to changes of metabolic function of gut microbiota. Lipopolysaccharides (LPS) produced by gut microbiota increased significantly, which may be a key factor in the subsequent liver effects.

**Conclusions:**

TiO_2_ NPs could induce slight hepatotoxicity at dose of 50 mg/kg after long-term oral exposure. The indirect pathway of the gut-liver axis, linking liver metabolism and gut microbiota, played an important role in the underlying mechanisms.

## Background

Titanium dioxide (TiO_2_) is a traditional common pigment used for whitening and brightening in paints, cosmetics, sunscreens, foods, pharmaceutical pills, and toothpastes [1]. Since the last decade, millions of tons of TiO_2_ was produced worldwide as a pigment, and the total production volume of TiO_2_ nanoparticles (TiO_2_ NPs) was estimated to be about 10,250 tons [2, 3]. Inside the panel of applications, these TiO_2_ NPs are used on a large scale as food and drug additives. Recently, two tests of food-grade TiO_2_ both suggested that approximately 36% of the particles are TiO_2_ NPs [4, 5]. A study also showed that over 40% of TiO_2_ particles in commercial gums is TiO_2_ NPs, which can leach out and be swallowed when chewing [6]. Due to its excellent photocatalytic activity, TiO_2_ NPs have also been widely used for self-cleaning surfaces and water/atmosphere purification [7–9], making it easy for TiO_2_ NPs to enter the environment and pose a risk for organisms and humans. Indeed, it has been estimated that the human dietary exposure dose of TiO_2_ NPs has reached 2.16 to100 μg/kg body weight per day (BW/d), and children were identified as having the highest exposures because the TiO_2_ content of sweets is higher than that of other food products [4, 10]. Meanwhile, one of the latest research studies detecting TiO_2_ particles in human post-mortem liver and spleen showed that more than 24% of TiO_2_ particles were NPs, and the authors emphasized that health risks due to oral exposure to TiO_2_ NPs are not paid enough attention [11].

Although bulk TiO_2_ was considered to be an inert and safe substance, existing studies have suggested that TiO_2_ NPs may be more toxic than traditional larger particles of TiO_2_ [12]. The reason for this is because NPs have higher biological activity than larger sized particles due to their larger surface area, they have improved penetration to cells, increased catalytic activity, and increased reactivity [13, 14]. The National Institute for Occupational Safety and Health (NIOSH) also supports this distinction by setting two separate occupational exposure limits for fine TiO_2_ particles and ultrafine TiO_2_ (< 100 nm). Thus, traditional knowledge on the safety of occupational and environmental exposure to TiO_2_ was challenged. Particularly, health risk due to oral ingestion of consumed TiO_2_ NPs in food products merits separate evaluations. Thus far, some studies have investigated the health effects of TiO_2_ NPs via oral exposure. The hepatotoxicity of TiO_2_ NPs has been reported by most studies, and the liver may be the target organ [15–19]. However, the underlying mechanism has not yet been clearly elucidated. Especially, attention should be given to the health risk of liver damage caused by long-term and low-dose dietary and environmental exposure to TiO_2_ NPs.

According to previous studies, when TiO_2_ NPs enter cells, they could directly cause a large increase in reactive oxygen species (ROS) by triggering respiratory burst and the ROS regeneration cycle [20], resulting in inflammation, mitochondria function and structure damage, genetic material damage, and then leading to cell autophagy, apoptosis, or necrosis. However, considering that only approximately 0.02 to 0.1% of TiO_2_ NPs could be absorbed through the digestive tract and the rest were excreted through feces [21–24], the proportion of organ damage caused by direct interaction of TiO_2_ NPs with cells should be very small. Meanwhile, it was reported that TiO_2_ NPs could have a significant impact on intestinal bacteria such as *Escherichia coli* under dark conditions by destroying the integrity of the cell membrane and causing osmotic stress [25]. A number of studies in recent years have demonstrated that alterations of gut microbiota can contribute to the pathogenesis of many disorders, including liver disease [26, 27]. Dietary or environmental factors can affect the composition of the intestinal microbiome and development of liver diseases, such as non-alcoholic fatty liver disease (NAFLD) and steatohepatitis (NASH) [28], possibly having a profound impact on host metabolism and health through intestinal-hepatic circulation and the gut-liver axis [29]. However, the health risk of liver disease from dietary or environmental exposure to nanomaterials (NMs) and its underlying mechanisms have not yet been reported. In the present study, we speculated that orally administered TiO_2_ NPs may interact with gut microbiota, thereby affecting the gut-liver axis and indirectly causing hepatotoxicity.

To test this hypothesis, we set out to investigate the influence of TiO_2_ NPs on gut microbiota and hepatic metabolism after oral exposure in rats. Through non-targeted metabonomics using high-performance liquid chromatography-mass spectrometry (HPLC-MS), we explored significantly changed liver metabolites and metabolic pathways. Structure and abundance changes of gut microbiota were analyzed by 16S rDNA sequencing analysis in stool samples. In addition, the metabolic capacities of gut microbiota were predicted and analyzed using the Tax4Fun software package. By using bioinformatics methods and detecting several typical metabolites of gut microbiota, we explored the potential role of the gut-liver axis in hepatotoxicity induced by oral administration of TiO_2_ NPs. We hope to provide clues for exploring and preventing environmental risk factors for liver disease.

## Results

### Physicochemical properties of TiO_2_ NPs

**Fig. 1:**
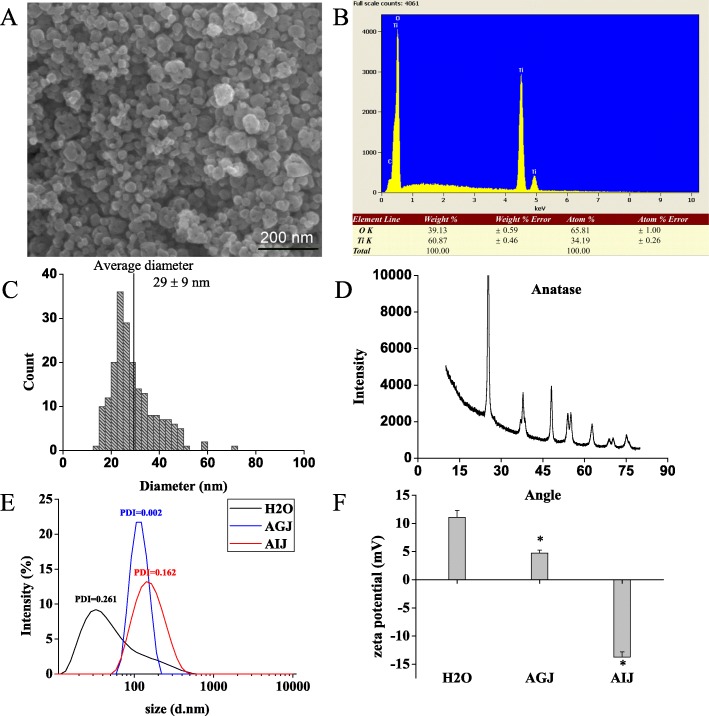
Characterization of TiO_2_ NPs. **a** The representative SEM image of TiO_2_ NPs. **b** The determination of element content of TiO_2_ NPs by EDX analysis. **c** Size distribution and average size of TiO_2_ NPs measured from SEM images. **d** The crystal analysis chart for TiO_2_ NPs by XRD. The hydrodynamic diameter (**e**) and zeta potential (**f**) of TiO_2_ NPs (1 mg/mL) in ultrapure water (H_2_O), artificial gastric juice (AGJ), and artificial intestinal juice (AIJ). Significant difference compared with the group of TiO_2_ NPs in H_2_O (∗*p* < 0.05). PDI: poly dispersity index

The majority of the TiO_2_ NPs used in this study were spherical anatase crystals with a purity of 99.90%. As shown in Fig. 1, the average size of the TiO_2_ NPs measured by SEM was 29 ± 9 nm, and the atomic ratio of Ti and O was 1:2. The measured BET specific surface area of the TiO_2_ NPs was 77.51 m^2^/g. To characterize TiO_2_ NPs in the exposure medium and rat gastrointestinal tract, the hydrodynamic diameter and zeta potential of TiO_2_ NPs (1 mg/mL) in ultrapure water (H_2_O), artificial gastric juice (AGJ), and artificial intestinal juice (AIJ) were tested. As shown in Fig. 1, it was clear that the hydrodynamic size of TiO_2_ NPs was larger in H_2_O, AGJ, and AIJ than their primary size, which was likely due to the aggregation and the adsorption of biomolecules. The Zeta potential of TiO_2_ NPs in AGJ and AIJ were opposite due to the different ionic strength and pH. These results suggested that TiO_2_ NPs tended to agglomerate to form larger particles in the gastrointestinal tract but the charges were different in the stomach and intestines.

### Hepatotoxicity induced by oral exposure to TiO_2_ NPs for 90 days

**Fig. 2:**
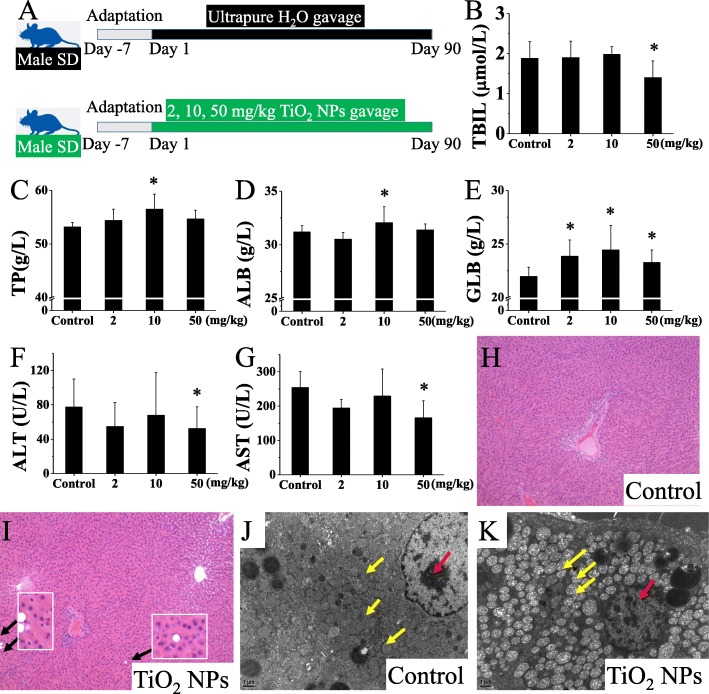
Hepatotoxicity induced by TiO_2_ NPs via oral exposure for 90 days. **a** Schematic diagram for oral administration of TiO_2_ NPs (0, 2, 10, and 50 mg/kg/d) in SD rats for 90 d. Hepatotoxicity was evaluated by serum biochemical indicators related to liver damage and histopathological observation. The serum levels of TBIL (**b**), TP (**c**), ALB (**d**), GLB (**e**), ALT (F), and AST (**g**) in rats were detected after TiO_2_ NPs treatment for 90 consecutive days. **h** and **i** were representative pathological images of HE staining under light microscopy (magnification: 20×). Fatty degeneration of hepatocytes, which appeared as fat vacuoles (black arrow), was evident in the 50 mg/kg BW TiO_2_ NPs treated group. **j** and **k** were representative TEM images of liver tissues (magnification: 8000×). Under TEM, it was observed that vacuolation of mitochondria (yellow arrows) around nuclei (red arrow) in hepatocytes was obvious in the 50 mg/kg BW TiO_2_ NPs treated group. Significant difference compared with the control group (∗ *p* < 0.05). TBIL: total bilirubin, TP: total protein, ALB: albumin, GLB: globulin, ALT: alanine aminotransferase, AST: aspartate aminotransferase

Figure 2 shows the hepatotoxicity induced by oral exposure to TiO_2_ NPs for 90 days. Some biochemical markers of liver injury were detected in serum, but also some markers contra-indicating liver injury. We found significantly increased levels of total protein (TP), albumin (ALB), and globulin (GLB) in TiO_2_ NP exposure groups (10 and 50 mg/kg BW) compared with the control groups. A significant increase in GLB was observed in the TiO_2_ NP exposure group even at a dose of 2 mg/kg BW. However, decreased levels of TBIL (total bilirubin), alanine aminotransferase (ALT), and aspartate aminotransferase (AST) in the TiO_2_ NP exposure group were also observed. Meanwhile, the representative pathological and TEM images of the liver tissue sections were also presented. Fatty degeneration of hepatocytes, which appeared as some fat vacuoles, was evident in the 50 mg/kg BW TiO_2_ NP treated rats. TEM images also showed vacuolation of mitochondria around nuclei in hepatocytes in the high-dose exposure group. These results indicated that subchronic oral exposure to 50 mg/kg BW/d TiO_2_ NPs induced significant hepatic toxicity in rats. Fatty degeneration and changes of mitochondria in hepatocytes suggested that energy metabolism may be disrupted.

### Changes of liver metabolism

**Fig. 3:**
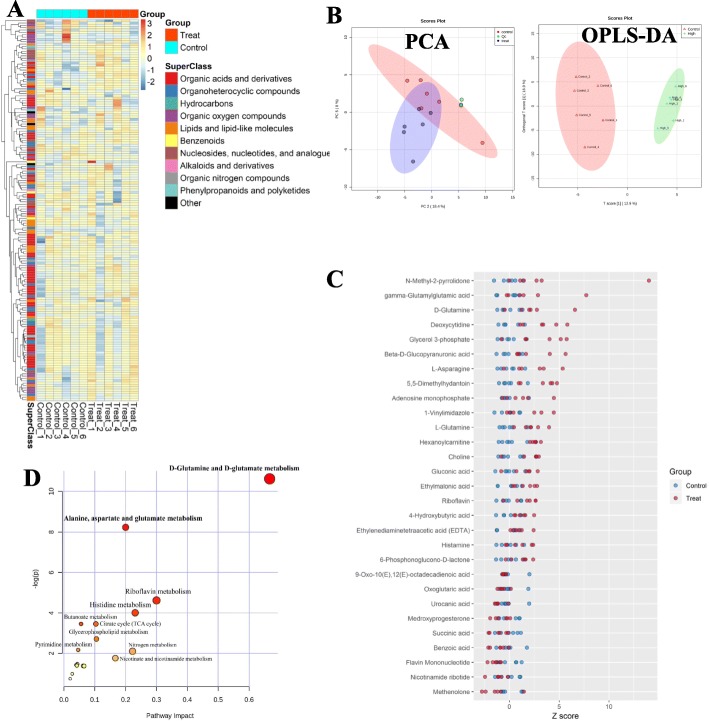
Effect of TiO_2_ NPs on liver metabolism analyzed by non-targeted metabonomics using HPLC-MS. **a** Heatmap of metabolite concentrations, which shows the different metabolites in liver tissues of rats in the control group and high-dose TiO_2_ NPs (50 mg/kg) treated group. Each row represents a metabolite, and each column represents a sample. The color of each grid represents the relative concentration of the metabolites in the corresponding sample. **b** PCA and OPLS-DA scoring maps of metabolites in the control and TiO_2_ NPs treated group as well as quality control (Qc) samples. **c** Z Score Map of differential metabolites between the liver samples in the control and TiO_2_ NPs treated group. Z score = (the sample concentration - average concentration of samples in the control group)/ standard deviation of sample concentrations in the control group. Each point represents a sample, blue as the control group and red as the treat group. **d** KEGG pathway analysis of differential metabolites. X-axis represents the Pathway Impact obtained by the out-degree centrality algorithm. The size of the point is related to the Pathway Impact. Y-axis represents the negative logarithm of the *p* value (−log(p)) obtained by the pathway enrichment analysis. The yellow-red color change of the point is positively related to the -log(p). The names of pathways are labeled in the graph with -log(p) > 2 or Pathway Impact > 0.1

As shown in Fig. 3a, the heatmap showed that there were differences in the expression of some metabolites among different groups. Principal component analysis (PCA) results showed that the principal component scores of three Qc samples were very close, indicating that the instrument was stable and the results obtained were reliable (Fig. 3b). And, a clear separation trend was shown between the TiO_2_ NPs exposure group and the control group in the PCA figure, suggesting that there was a significant change in metabolites between different groups. The score map of the orthogonal projection to latent structure discriminant analysis (OPLS-DA) model effectively distinguishing the samples of different groups further validated these results (Fig. 3b).

As shown in Fig. 3c, a total of 29 metabolites differentially expressed between groups were screened from 263 metabolites. The standardized Z-scores of these 29 metabolites are presented. The statistical analysis results are shown in Additional file 1: Figure S3, using a V-plot of the OPLS-DA model and p (corr)1 > 0.5 as the criterion. Among them, the concentrations of 9 metabolites including benzoic acid, succinic acid, and oxoglutaric acid decreased significantly compared with the control group, and 20 metabolites including D-glutamine, glycerol 3-phosphate, and choline increased significantly (detailed information of differential metabolites is shown in Additional file 1: Table S2).

Pathway topology analysis found that the D-glutamine and D-glutamate metabolic pathway (*Holm P* = 0.002, FDR = 0.002, pathway impact = 0.67) and the metabolic pathway of alanine, aspartate, and glutamate (*Holm P* = 0.021, FDR = 0.011, pathway impact = 0.20) significantly changed in the TiO_2_ NPs exposure group (Fig. 3d and Additional file 1: Table S3).

### Oxidative stress and inflammatory response

Several markers of oxidative stress, including the levels of reduced (GSH) and oxidized (GSSG) glutathione, MDA, and the activity of GSH-Px, SOD, were measured in liver tissues of rats after oral exposure to TiO_2_ NPs for 90 days (Table 1). Decreased GSH and increased GSSG were found in rats after TiO_2_ NPs exposure (10 and 50 mg/kg BW) compared with that in the control group. The ratio of GSH/GSSG decreased significantly, which indicated that the redox balance was disrupted. TiO_2_ NPs also caused accumulation of lipid peroxidation (MDA) and increased activity of GSH-Px and SOD. The changes of these oxidative stress biomarkers indicated that oxidative stress state was induced by subchronic oral exposure to 10 and 50 mg/kg BW TiO_2_ NPs. Meanwhile, we evaluated inflammatory status of rats by quantifying the concentrations of inflammatory cytokines in serum. We observed increased concentration of IL-1α, IL-4, and TNF in the serum of rats treated with 50 mg/kg TiO_2_ NPs for 90 days. Obviously, inflammatory response was induced by oral exposure to 50 mg/kg BW TiO_2_ NPs.

**Table 1:** Effect of TiO_2_ NPs on oxidative stress biomarkers in the liver tissue and inflammatory cytokines in serum of rats after oral exposure for 90 days

	Control	TiO_2_ NPs treated doses (mg/kg BW)		
		2	10	50
GSH (nmol/mg protein)	19.81 ± 4.60	21.20 ± 5.62	18.51 ± 3.80*	17.28 ± 3.48*
GSSG (nmol/mg protein)	8.82 ± 1.27	9.69 ± 1.53	9.45 ± 1.31*	9.28 ± 2.13*
GSH/GSSG	2.23 ± 0.23	2.18 ± 0.44	1.95 ± 0.22*	1.88 ± 0.23*
GSH-Px (mU/mg protein)	161.18 ± 11.43	192.84 ± 57.97	205.05 ± 40.47*	210.04 ± 82.34*
MDA (nmol/mg protein)	3.40 ± 2.21	3.02 ± 2.49	11.82 ± 17.49	43.80 ± 27.51*
SOD (U/mg protein)	10.56 ± 1.06	12.62 ± 4.76	13.64 ± 2.93*	14.84 ± 6.30
IL-1α (pg/mL)	11.48 ± 2.34	10.35 ± 0.64	11.40 ± 1.23	13.41 ± 2.54*
IL-4 (pg/mL)	1.35 ± 0.11	1.40 ± 0.18	1.42 ± 0.13	1.83 ± 1.38*
TNF (pg/mL)	13.69 ± 1.57	13.43 ± 2.80	14.36 ± 2.30	13.51 ± 1.43*

* Significant difference compared to the control group (*p* < 0.05). *NPs* nanoparticles, *BW* body weight, *GSH* reduced glutathione, *GSH-Px* glutathione peroxidase, *GSSG* oxidized glutathione, *MDA* malondialdehyde, lipid peroxidation products, *SOD* superoxide dismutase, *IL* interleukin, *TNF* tumor necrosis factor

### Changes of gut microbiota

**Fig. 4:**
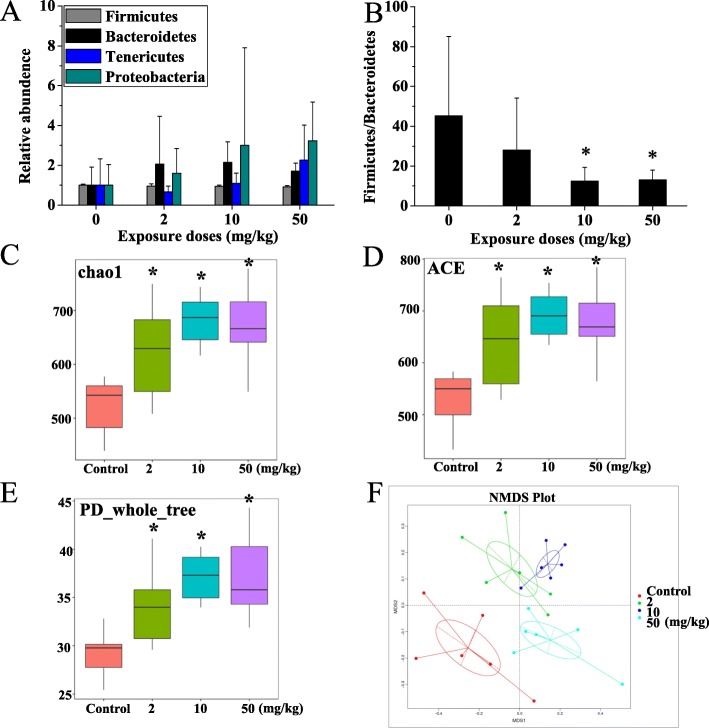
Effect of TiO_2_ NPs on gut microbiota after oral administration for 90 days. **a** Top four relative abundance of bacteria at phylum-level. **b** Ratio of relative abundance between *Firmicutes* and *Bacteroidetes*, which may be closely related to adverse health effects of hosts. Comparison of Alpha Diversity of gut microbiota between different groups using Chao1 (**c**), ACE (**d**), and PD_Whole_Tree (**e**). **f** Comparison of Beta Diversity of gut microbiota between different groups using non-linear model NMDS method based on OTUs information in samples. * represents statistical difference (*p* < 0.05)

As shown in Fig. 4, it was observed that the diversity of gut microbiota in rats increased with the increase of the exposure doses of TiO_2_ NPs. The top four bacteria as far as relative abundance at the phylum level were *Firmicutes*, *Bacteroidetes, Tenericutes*, and *Proteobacteria*. We found that the ratio of relative abundance between *Firmicutes* and *Bacteroidetes* was significantly decreased in the TiO_2_ NPs exposure groups, which may be closely related to adverse health effects of hosts. The structure and composition of gut microbiota communities at class, order, family, and genus levels (top 10) are also shown in Additional file 1: Figure S4. The comparison of alpha (α) and beta (β) diversity between groups is shown in Fig. 4c-f. The Chao1 and ACE scores and index of PD_whole_tree of low-, medium-, and high-dose groups were significantly higher than those of the control group, which suggested that the species richness of gut microbiota in rats increased after oral administration of TiO_2_ NPs. Non-parametric NMDS analysis showed that the samples in the same group were aggregated, and the samples in different groups were distinguished clearly (stress = 0.173 < 0.200), suggesting that the difference of gut microbiota composition among groups was obvious.

**Fig. 5:**
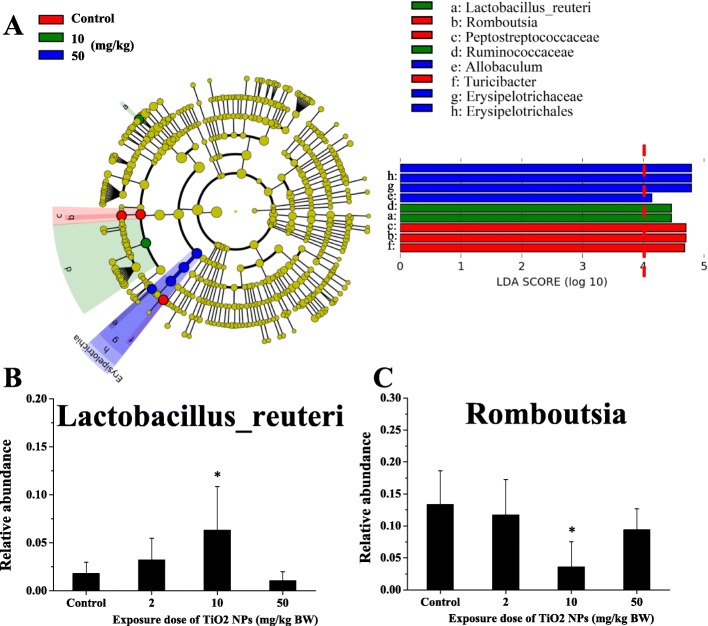
Differential gut microbiota between the samples in the control and TiO_2_ NPs treated group. **a** Evolutionary branch diagram of differential gut microbiota. The circle radiated from inside to outside represents the classification level from phylum to species. Each small circle at different classification levels represents a kind of bacteria at the corresponding level. The diameter of the small circle is positively related to the relative abundance. Coloring principle: bacteria with no significant difference between groups colored by yellow; bacteria with enrichment (LDA > 4) in the control group, medium and high dose of TiO_2_ NPs treated group colored by red, green, and blue, respectively. The relative abundance of *Lactobacillus_reuteri* (**b**) and *Romboutsia* (**c**) were significantly changed in the TiO_2_ NPs treated group, compared with the control group. * represents statistical difference (*p* < 0.05)

LefSe analysis was used to identify differentially expressed strains among different groups. The branching evolutionary map with the different strains labeled and the primitive data histogram of relative abundance of different strains are shown in Fig. 5a. A total of nine differentially expressed strains at different taxonomic levels (LDA score > 4) were obtained. After merging and retaining the lowest taxonomic level of duplicated results, six different strains were reserved, all belonging to *Firmicutes*. They were *Bacilli-Lactobacillales-Lactobacillaceae-Lactobacillus-Lactobacillus_reuteri, Clostridia-Clostridiales-Peptostreptococcaceae-Romboutsia, Clostridia-Clostridiales-Ruminococcaceae, Erysipelotrichia-Erysipelotrichales-Erysipelotrichaceae-Allobaculum, Erysipelotrichia-Erysipelotrichales-Erysipelotrichaceae-Turicibacter,* and *Erysipelotrichia-Erysipelotrichales-Erysipelotrichaceae*, respectively. The results of comparisons between exposure groups and the control group of different strains are shown in Fig. 5b-c. Compared with the control group, *Lactobacillus_reuteri* (*L. reuteri*) in the medium-dose group increased significantly (*p* = 0.038), whereas *Romboutsia* decreased significantly (*p* = 0.008).

The metabolic pathway changes of gut microbiota in fecal samples were predicted by Tax4Fun tool. The predicted results showed that 258 KEGG metabolic pathways were involved in the gut microbiota, and the highest abundance was in the ATP binding and transporter metabolic pathway. Further, three metabolic pathways were significantly activated and increased in the exposed groups compared with the control group, including the glycosaminoglycan degradation pathway, the fat digestion and absorption pathway, and the systemic lupus erythematosus pathway (Additional file 1: Table S4).

### Changes of LPS and SCFAs

**Fig. 6:**
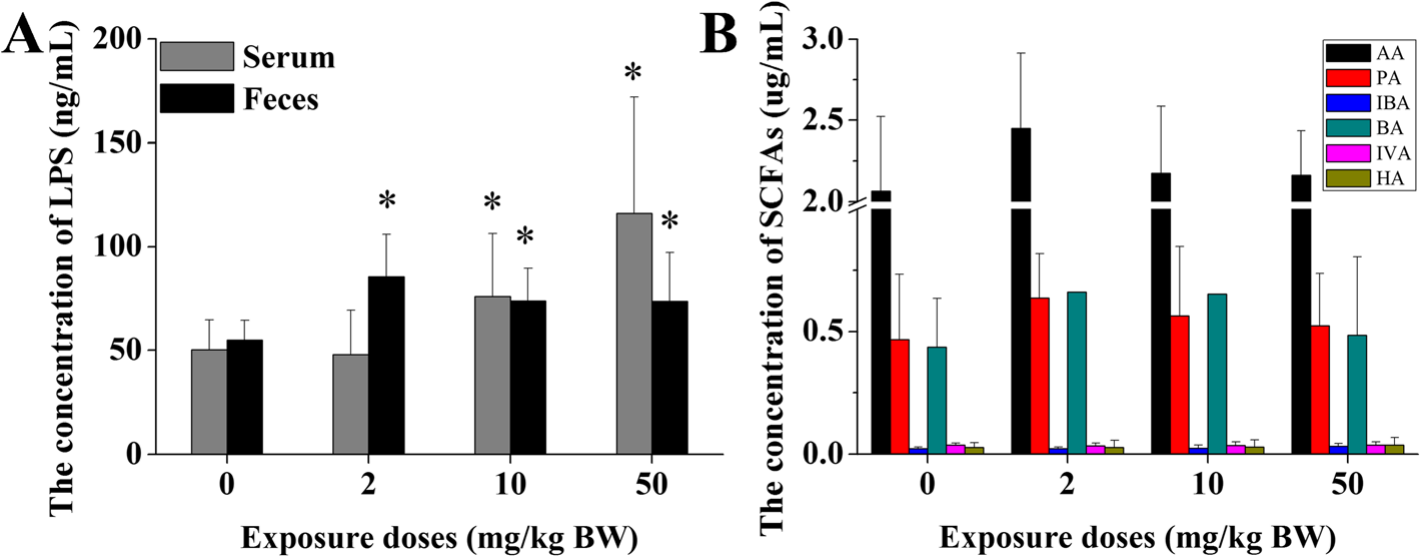
Effect of TiO_2_ NPs on the content of LPS and SCFAs. **a** LPS concentrations in the serum and fecal samples increased significantly in the TiO_2_ NPs exposure groups compared with the control group. **b** SCFAs, including acetic acid (AA), propionic acid (PA), isobutyric acid (IBA), butyric acid (BA), isovaleric acid (IVA), and hexanoic acid (HA), did not change significantly after exposure to TiO_2_ NPs in the feces. Significant difference compared with the control group (∗ *p* < 0.05). LPS: lipopolysaccharides; SCFAs: short-chain fatty acids

As shown in Fig. 6, LPS content in the feces increased significantly in the TiO_2_ NPs exposure groups compared with the control group. LPS is a major component of the outer membrane of gram-negative bacteria, and its mutation or removal will result in death. The increase of LPS content in the feces should be the result of the changes of gut microbiota. SCFAs, known as volatile fatty acids, play important roles in the metabolism of different organs in the human body. The type and quantity of SCFAs mainly depend on the composition of gut microbiota, digestion time, and host microbial generation. We found that six SCFAs in the feces, including AA, PA, IBA, BA, IVA, and HA, did not change significantly after exposure to TiO_2_ NPs.

## Discussion

In the present study, we focused on the effects of TiO_2_ NPs on gut microbiota and hepatic metabolism in rats and explored the potential role of the gut-liver axis in hepatotoxicity induced by the oral administration of TiO_2_ NPs. First, we confirmed that hepatotoxicity was significant in rats after repeated (50 mg/kg BW) oral exposure to TiO_2_ NPs for 90 days (subchronic), through apparently altered biochemical and pathological results (Fig. 2). The hepatotoxicity of TiO_2_ NPs after a longer oral exposure should call attention to its human and environmental health hazard. Summarizing previous literature, the liver may be the most sensitive organ of toxicity induced by TiO_2_ NPs via oral exposure. Wang et al. found that the significant changes of serum biochemical parameters (ALT/AST, LDH) and pathology (hydropic degeneration around the central vein and the spotty necrosis of hepatocytes) of the liver indicating the obvious hepatotoxicity were induced by acute oral exposure of TiO_2_ NPs (25 and 80 nm) [18]. But, the dose used in that study (5000 mg/kg BW) was too large to be correlated with actual human exposure. Shukla et al. [30] observed a significant alteration in the level of hepatic enzymes and liver histopathology at an oral dose of 100 mg/kg TiO_2_ NPs for 14 days. High accumulation of TiO_2_ NPs in the liver tissue causing DNA damage and apoptosis through the intrinsic pathway was considered to be the main toxic mechanism. Two subacute toxicity studies have also shown that the liver may be the target organ of TiO_2_ NPs-induced toxicity [17, 19]. The liver damage observed in mice treated with higher doses of TiO_2_ NPs was likely associated with the damage of the hemostasis blood system and immune response [17]. The pathway and mechanism of hepatotoxicity induced by TiO_2_ NPs may be different under different exposure doses and time.

In theory, TiO_2_ NPs could induce hepatotoxicity through direct and indirect pathways. Direct pathway refers to the accumulation of TiO_2_ NPs in the liver after absorption by the digestive tract, and thereby impairing the liver tissue directly. Previous toxicokinetic studies showed that TiO_2_ NPs could be transported to the liver, spleen, kidney, lung, and brain in mice after acute exposure at a very high dose of 5 g/kg body weight [17, 18, 31]. The liver was found to be the main target tissue of accumulation of Ti, followed by spleen and lung [24]. Later researches, however, suggest that the oral absorption of TiO_2_ NPs is dose- and time-dependent [32]. Cho et al. [22] used lower doses (520.8, 1041.5, and 2083 mg/kg BW) and longer exposure time for 13 weeks, but found extremely low absorption of TiO_2_ NPs. In the present study, we evaluated the accumulation of TiO_2_ NPs in the liver by detecting Ti element, but did not find a significant increase of TiO_2_ NPs in the exposure group (Additional file 1: Figure S5). It indicated that the toxicokinetics of TiO_2_ NPs after longer exposure time at lower doses may be quite different from that after short-term and high-dose exposure, and direct pathway likely isn’t the main mechanism of hepatotoxicity.

In view of the fact that most orally ingested TiO_2_ NPs mainly acted in the intestine and its antimicrobial activity, it is reasonable to speculate that it will affect gut microbiota. Originally, TiO_2_ NP was reported to be a unique antimicrobial compound due to its ability to disrupt bacterial cell walls and cause cell death by producing ROS in the presence [33] and absence [25] of light. And, the TiO_2_ NPs with anatase crystal structure and smaller particle size produced higher content of intracellular ROS and MDA, in line with their greater antibacterial effect [34]. The results of 16rDNA sequencing analysis confirmed that the composition of gut microbiota was significantly affected by TiO_2_ NPs. Previously, Waller et al. [35] also found that TiO_2_ NPs (25 nm) could rapidly remodel the composition of intestinal bacteria in vitro and change the relative abundance of intestinal bacteria such as *Firmicutes* and *Proteobacteria*, using the model colon reactor. In the present study, *Lactobacillus reuteri* (*L. reuteri*) significantly increased, and *Romboutsia* significantly decreased in rats after oral exposure to TiO_2_ NPs for 90 days. We also found an increase in the diversity of the gut microbiota in TiO_2_ NPs treated rats, which was associated with positive effects on health by others. Higher gut microbiota diversity was reported to be associated with lower arterial stiffness and increased circulating levels of the anti-oxidant indoleproprionic acid in women [36, 37]. The reason for this positive effect in this study is still not clear. Maybe it is a compensation. *L. reuteri* is a kind of *Bacteria-Firmicutes-Bacilli-Lactobacillales-Lactobacillaceae-Lactobacillus* (classification) and one of the most widely used probiotics, which can produce a variety of metabolic molecules, stabilizing the intestinal environment and preventing the migration and expansion of opportunistic pathogens as well as reducing the inflammatory reaction [38]. *Romboutsia* belongs to *Bacteria-Firmicutes-Clostridia-Clostridiales-Peptostreptococcaceae*, which is a common intestinal bacteria and has been found to be highly related to body energy metabolism and very sensitive to bile salts [39]. Changes in the structure and abundance of gut microbiota would affect their metabolic function.

Three metabolic pathways for gut microbiota changes were predicted by the Tax4Fun tool in this study, including glycosaminoglycans (GAGs) degradation, fat digestion and absorption, and systemic lupus erythematosus (SLE). They were all upregulated in the TiO_2_ NPs exposure group. The upregulation of the GAGs degradation metabolic pathway in gut microbiota may be related to the oxidative stress-mediated inflammatory response and cell damage induced by TiO_2_ NPs. Studies have shown that GAGs can regulate LPS-mediated inflammation, protect cells under oxidative stress, and reduce the toxicity of pathogenic microorganisms [40]. This may correspond to our subsequent results that TiO_2_ NPs induced the increase of LPS content in feces (Fig. 6). Gut microbiota can provide enzymes to participate in the hydrolysis of fat and regulate the intestinal decomposition and absorption of fatty acids. Martinez-Guryn et al. [41] transplanted gut microbiota of obese mice induced by a high-fat diet into the small intestine of aseptic mice. It was found that the absorption of fatty acids increased in aseptic mice and the genes related to fat absorption in proximal intestinal epithelial cells were significantly upregulated. Although we did not find any significant changes in the SCFAs detected, the changes of fat digestion and absorption metabolic pathway of gut microbiota observed in this study were mutually corroborated with fatty degeneration of hepatocytes observed by pathology. SLE is an autoimmune disease caused by an immune system disorder. Previous studies have reported that there may be a close relationship between the differential intestinal bacteria *L. reuteri* observed in the present study and SLE diseases [42, 43]. They suggested that the elevation of *L. reuteri* may be closely related with the host inflammation and anti-inflammatory state.

Indeed, inflammatory responses and oxidative stress had been originally thought to be the mechanism where oral exposure to the TiO_2_ NPs induced toxicity. Trouiller et al. [44] observed elevated expression of inflammatory cytokines such as TNF-α, IFN-γ, and IL-8 in the blood of mice after oral intake of TiO_2_ NPs at 100 mg/kg BW for 5 days. They suggested that the genotoxicity in vivo in mice induced by TiO_2_ NPs may be mainly associated with the inflammation and/or oxidative stress, which was called a secondary genotoxic mechanism. Afterward, several publications confirmed that the genotoxicity of most NMs is likely to be associated with indirect consequences of inflammation and generation of oxidative species by inflammatory cells (neutrophils and macrophages) [45, 46]. In addition, Duan et al. [17] found that the liver function damage observed in mice treated with 125 and 250 mg/kg BW TiO_2_ NPs (5 nm) for 30 days is likely associated with the damage of the hemostasis blood system and immune response. Therefore, we speculated that hepatotoxicity induced by TiO_2_ NPs via long-term oral exposure was mainly through indirect pathways, such as oxidative stress and inflammatory responses. In fact, we did find obvious oxidative stress and inflammatory responses in rats after oral administration of TiO_2_ NPs for 90 days (Table 1). However, the original sites of oxidative stress and inflammation induced by TiO_2_ NPs and their more advanced mechanism, such as how to cause them, remain unclear.

Toxic agents related to gut microbiota, which can be transported through the intestinal-hepatic circulation, can cause a series of adverse effects including activation of oxidative stress and inflammation as well as endoplasmic reticulum (ER) stress and mitochondrial dysfunction in the intestine or liver. Thus, the role of the gut-liver axis provides insights into the mechanism of hepatotoxicity caused by these inert NMs. Modifications to the gut microbiota can provide signals through the intestine and bacterial products, as well as hormones produced in the bowel that affect metabolism at different levels including the liver [28]. Therefore, metabonomics analysis of liver tissue was conducted to further explore the liver metabolic changes. We observed the disturbance of metabolites related to energy metabolism in this study, which may be the key to pathological changes of fatty degeneration in hepatocytes in TiO_2_ NPs exposure groups. Other studies also indicated that liver lipid metabolism disorder should be one of the obvious toxic effects of TiO_2_ NPs [47, 48]. Importantly, the link between the gut microbiota and adipose tissue has been recently identified. It has been shown that LPS acts as a master switch to control adipose tissue metabolism [49, 50]. Meanwhile, the increased choline and 9-Oxo-10 (E), 12 (E) -octadecadienoic acid (a derivative of linoleic acid) (Fig. 3c) were reported to be one of the causes of liver lipid metabolism disorder [51, 52]. Choline can synthesize phosphatidylcholine and assemble into very-low-density lipoprotein to transport triglycerides from the liver to other tissues. In addition, two other differentially expressed metabolites including gluconic acid (a metal chelating agent) and beta-D-glucopyranuronic acid (a stable conformation of glucuronic acid) (Fig. 3c) may also mediate the interference of TiO_2_ NPs on liver lipid metabolism [53]. These two metabolites can bind to exogenous compounds and increase their polarity and water solubility, so that the compounds can be eventually discharged from the kidney. As an important means of eliminating exogenous compounds, the increase of these two metabolites should be the compensatory regulation of the body.

Compared with the control group, the differential metabolites in the TiO_2_ NPs exposure group were significantly enriched in two metabolic pathways. The first was the D-glutamine_D-glutamic acid metabolic pathway, which may be very important for toxic mechanisms of TiO_2_ NPs on oxidative stress and disordered glucose homeostasis reported in the past. Glutamic acid is a kind of non-essential amino acid that participates in the synthesis of various metabolites and regulation of multiple signaling pathways [54]. Glutamic acid can produce GSH, an antioxidant, in response to oxidative damage. It has been widely reported that TiO_2_ NPs could destroy the oxidative/antioxidative balance by increasing ROS. The significant changes in the glutamine_glutamic acid metabolic pathway observed in this experiment may be due to resistance to increased ROS produced by TiO_2_ NPs. Glutamate dehydrogenation can promote insulin secretion, which leads disordered glucose metabolism [55]. Previous studies have found that the oral administration of TiO_2_ NPs could cause disordered glucose homeostasis in rats [56, 57]. A positive relationship between insulin resistance and the concentration of glutamine and ratio of glutamine/glutamic acid had been proved in two cohorts [58].

The second significantly enriched metabolic pathway in the present study was the alanine_aspartic acid_glutamic acid metabolic pathway, which is closely related to energy-related metabolism [59]. Previous in vitro and in vivo studies have shown that the energy-related metabolic pathway could be affected by the exposure of TiO_2_ NPs [60, 61]. Chen et al. [60] reported that TiO_2_ NPs increased ROS of mitochondria and decreased ATP production in macrophage cells (RAW). The TCA cycle tracking 13C isotopic labeled glutamine by metabolic flow technology was significantly downregulated in a dose-dependent manner. Mitochondria containing a variety of redox enzymes during the TCA cycle is the key subcellular organelle of energy metabolism. TiO_2_ NPs could alter mitochondrial membrane potential and membrane integrity and affect the activity of key enzymes of the electron transfer chain in hepatocytes [62]. This also could correspond to the mitochondria damage in liver tissues we observed under TEM (Fig. 2).

**Fig. 7:**
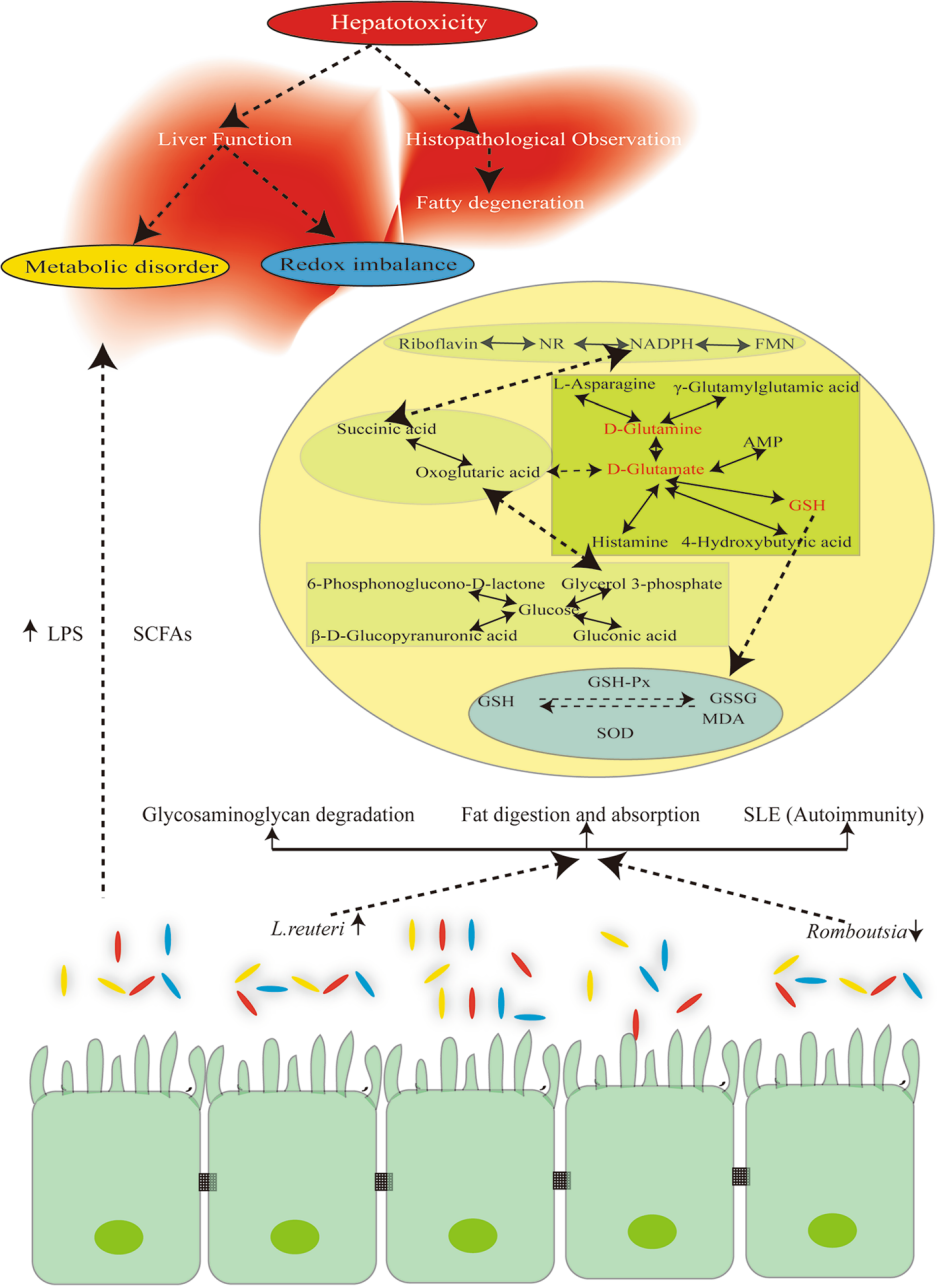
Interaction network of gut microbiota, liver metabolism, and hepatotoxicity after oral administration of TiO_2_ NPs. The changes of gut microbiota represented by increased *L. reuteri* and decreased *Romboutsia* led to the changes of intestinal metabolic function. The glycosaminoglycan degradation, fat digestion and absorption, and autoimmune-related metabolic pathways of gut microbiota increased significantly after exposure to TiO_2_ NPs. In addition, LPS produced by gut microbiota increased significantly, which may be a key factor in the subsequent liver effects. Hepatotoxicity manifested as changes in liver function and. Metabolic disorders and redox imbalances should be the main causes of liver function changes. In the complex metabolic network of the liver, glutamate, glutamine and glutathione may be the key metabolites leading the generation of energy-related metabolic disorders and imbalance of oxidation/antioxidation

According to our results, the alteration of the gut-liver axis and co-metabolism between gut microbiota and the host liver may be the main mechanism for hepatotoxicity induced by oral administration of TiO_2_ NPs, which was summarized and shown in Fig. 7. This may be suitable for most cases of liver damage caused by oral intake of NMs. Because NMs generally have higher surface activity, they can easily interact with gut microbiota. But what needs to be mentioned is that NMs only account for a small proportion of human dietary intake. This effect may be far away from the real situation of human body, considering the interference from the rest of human diet. Nevertheless, this provides a scientific clue for exploring the mechanisms of the biological effects of NMs after oral exposure and for further finding measures to reduce the dietary and environmental health risks of NMs.

## Conclusion

In conclusion, TiO_2_ NPs could induce slight hepatotoxicity at dose of 50 mg/kg BW/d after subchronic oral exposure. Various differential liver metabolites and intestinal bacterial species demonstrated that energy-related metabolic disorders and an imbalance of oxidation/antioxidation may be primarily responsible for the liver damage. The indirect pathway of the gut-liver axis, linking liver metabolism and gut microbiota, may play an important role in the underlying mechanisms. Attention should be paid to the health hazard of liver injury and gut microbiota disturbance caused by dietary and environmental exposure to NMs.

## Methods

### Nanoparticle characterization

The TiO_2_ NPs were purchased from Shanghai Macklin Reagent Co. Ltd., China. The size and shape of the particles were characterized by scanning electron microscopy (SEM, Nova, Tecnai F30, FEI Company, Oregon, USA). Energy dispersive X-ray spectroscopy (EDS, Nova_NanoSEM430, FEI Company, Oregon, USA) was used to measure the ratio of Ti to O atoms. The purity of the particles was analyzed by detecting the content of Ti element using inductively coupled plasma mass spectrometry (ICP-MS, IRIS Advantag, TJA, Franklin, MA, USA). The crystal structure of the particles was identified by X-ray powder diffractometry (XRD, PANalytical’s X’Pert PRO, X’Celerator, EA Almelo, Netherlands). The specific surface area (SSA) of the particles was measured according to the Brunauer–Emmett–Teller (BET) method (Quantachrome, Autosorb 1, Boynton, FL, USA).

The artificial gastric juice (AGJ, pH = 1.2) was prepared using 10 g/L pepsin (3800 units/mg) and 45 mmol/L HCl. The artificial intestinal juice (AIJ, pH = 6.8) was made with 10 g/L trypsin (2500 units/mg) and 6.8 g/L KH_2_PO_4_. The pH was adjusted to 6.8 using 0.1 mol/L NaOH. After TiO_2_ NPs were dispersed in ultrapure water (H_2_O), AGJ or AIJ to obtain a final concentration of 1 mg/mL TiO_2_ NPs, the suspensions were supersonicated for 15 min to break up aggregates. The particle hydrodynamic diameters and Zeta potentials were tested using the ZetaSizer Nano ZS90 (Malvern Instruments Ltd., Malvern, UK).

### Animals and experimental design

Three-week-old healthy Sprague-Dawley (SD) rats were bred and supplied by the Department of Laboratory Animal Science, Peking University Health Science Center. The rats were fed a commercial pellet diet and deionized water ad libitum and were kept in plastic cages at 20 °C ± 2 °C and 50 to 70% relative humidity with a 12:12-h light-dark cycle. After 1 week of acclimation, the rats were weighed and randomized into experimental and control groups, with six male rats in each treatment group.

All experimental rats were provided humane care. The study was conducted in accordance with the guidelines of European Union Directive 2010/63/EU for animal experiments, and received approval from the Peking University Institutional Review Board (Approval number: LA2017073). The design of animal experiment refers to OECD Guidelines for the Testing of Chemicals No. 408 Repeated Dose 90-Day Oral Toxicity Study in Rodents.

The TiO_2_ NPs were dispersed in ultrapure water and sonicated for 15 min. To obtain homogenized suspension, the particle suspension was vortexed before every use. Suspensions of TiO_2_ NPs (0, 2, 10, 50 mg/kg BW) were administered to rats via oral gavage in a volume of 1 mL daily for 90 consecutive days. The intragastric doses of TiO_2_ NPs for rats were selected based on the oral intake of TiO_2_ NPs for children [4, 10], using 100 as safety factor.

The symptoms and mortality were observed and recorded daily throughout the entire duration of exposure up to 90 days. The body weight of rats was assessed every 7 days, and the food intake of rats was recorded every 3 to 4 days. During the experiments, no significant changes in the body weight and food intake of the exposed rats were found (Additional file 1: Figure S1, S2), and no mortality was observed. After 90 days, rat feces were collected and quickly transferred and stored in an − 80 °C refrigerator. Then, animals were weighed and euthanized. The blood samples were collected from the abdominal aortic vasculature. Serum was obtained by centrifuging blood at 3000 rpm (1500 g) for 10 min. The liver tissues were harvested and weighed.

### Measurement of blood biochemical parameters and histopathological analysis related to hepatic damage

The serum levels of alanine aminotransferase (ALT), aspartate aminotransferase (AST), total bilirubin (TBIL), total protein (TP), albumin (ALB), and globulin (GLB) were assayed to evaluate hepatic damage. All biochemical assays were performed using a clinical automatic chemistry analyzer (Type AU400, Olympus, Japan).

For pathological studies, all histopathological examinations were performed using standard laboratory procedures. The liver tissues were embedded in paraffin blocks, then sliced into 5-μm slices and placed onto glass slides. After hematoxylin–eosin (HE) staining, the slides were observed and the photos were taken using an optical microscope (OlympusBX50, Moticam 2306, Japan). The pathologist who performed the observation and analysis was blinded of the treatment groups and dosing regimens. For TEM observation, the liver tissues were cut up into small pieces (1 mm^3^) and immediately fixed in 2.5% glutaraldehyde (pH 7.4) overnight. Then, the samples were treated according to the general protocols for TEM study. The ultra-thin sections (70–100 nm) were stained with lead citrate and uranyl acetate. The specimens were examined using JEOL JEM-1400 electron microscopy.

### Liver metabonomic analysis

The method for liver metabonomic analysis refers to the protocol of Want et al. [63].

- Homogenate of liver tissue and sample preparation.

30.0 mg of liver tissue was added to 900 μl pre-cooled methanol/water (1:1) solution. Then it was homogenized at 30000 rpm on ice for 30 s (Homogenized for 10 s, cooled for 30 s, repeated three times) and blended by a vortex for 20 s and stored at − 20 °C overnight. And then, the homogenate was centrifuged (16,000 g, 4 C) for 10 min and the supernatant was taken. The supernatant was dried and concentrated in a low temperature vacuum concentrator and for 4 h.

Re-suspension was carried out by 200 μl methanol/water (1:1) solvent before analysis. Meanwhile, 20 μl of each sample was taken and then divided into three parts after gentle mixing as quality control (Qc) samples, which were prepared for technical repetition to evaluate the stability and repeatability of the experimental instruments and methods.

(b) Non-targeted metabonomics analysis using HPLC-MS

Ultra High Performance Liquid Chromatography-Q-Exactive Orbitrap-High-resolution Mass Spectrometry System (UPLC-QEMS, U3000, Thermo, USA) was used for non-targeted metabolomics analysis. The samples were randomly injected after disruption of the order to control the possible impact of instrumental stability fluctuation. Three Qc samples were analyzed before experimental samples, after half of all samples and after all samples, respectively. The QEMS was equipped with an electrospray ionization source (ESI). Fragmentation was achieved by high-energy collision dissociation (HCD). The normalized collision energies were 15, 30 and 45 eV, respectively. The results were measured by positive ion mode and negative ion mode. The mass scanning range was 50–1100 m/z, and the total scanning resolution of parent ions (MS) was 60 K.

(c) Analysis and annotation of mass spectrometry data

The original result file obtained by instrument analysis (.raw format file, positive and negative ion mode data) is imported into Compound Discoverer 3.0 software (Thermo Fisher Scientific, USA) for peak alignment, deconvolution, noise filtering, mass-charge correction and baseline correction. The parameters are set as follows: retention time (RT) < 0.2 min; signal-to-noise ratio (SNR) > 3; DDA mode was used to analyze secondary ion mass spectrometry (MS_2_); signal intensity > 500,000 included in the analysis; the filling gap algorithm was used to extract and fill the peaks (More parameters for metabolite identification by Compound Discoverer software were shown in Additional file 1: Table S1).

The annotation and identification of metabolites were carried out through software-related mzCloud database and mzVault database. The peak area was used as the relative concentration for subsequent analysis. The data containing metabolite identification results and peak area were pretreated. The relative concentration of metabolites was completely clustered using Euclidean distance, and the Heatmap was drawn to show the difference of the concentrations of metabolites in each sample. Principal Component Analysis (PCA) was used to reduce the dimension of the original data and observe the difference trend and potential outlier value of samples. Qc samples were also included in PCA analysis and scoring map drawing. The stability of the instrument in the process of analysis was investigated by the aggregation of Qc samples in PCA scoring map. The closer the aggregation of Qc samples, the better stability of the instrument. Using the orthogonal projection to latent structure discriminant analysis (OPLS-DA) model to screen biomarkers that change after TiO_2_ NPs exposure, the OPLS-DA model score maps were drawn by the first predictive component (T score [1]) and the first orthogonal component (Orthogonal T score [1]). Through Simica-P. software (V14.1, Umetrics, Sweden), the permutation test of OPLS-DA model is performed to verify the stability of OPLS-DA model. Then V-Plot was draw according to the covariance (p1) and reliability (p(corr)1) of the first principal component of each variable in OPLS-DA model, and the metabolite with absolute value of p(corr)1 greater than 0.5 in V-Plot is selected as differential metabolite. Z Score-plot is drawn to visualize the distribution of different metabolites among groups through *ggplot2* packages in R. Z-score = ((Metabolite concentration - mean of metabolite concentrations in control group) / standard deviation of metabolite concentrations in control group).

After obtaining the differential metabolites, KEGG metabolic pathway was analyzed by Pathway Analysis function module in Metaboanalyst 4.0 website. Pathway topology analysis was conducted by using the pathway data in *Rattus norvegicus* pathway libraries (including 81 metabolic pathways), and hypergeometric test in over representation analysis was used to test the significance of metabolic pathway enrichment, and the out-degree centrality is used as a criterion to measure the impact of different metabolites on pathway. The significant changed pathway was determined by adjusted *Holm P* < 0.05 through Holm method (also known as step-down Bonferroni method), false discovery rate (FDR) < 0.05, and pathway impact greater than 0.10.

### Detecting oxidative stress biomarkers and inflammatory cytokines

Oxidative damage to the liver following repeated TiO_2_ NPs exposure was evaluated by the levels of reduced (GSH) and oxidized (GSSG) glutathione, glutathione peroxidase (GSH-Px), lipid peroxidation products (malondialdehyde, MDA), and superoxide dismutase (SOD) in tissue homogenates, which were tested using commercial kits (Nanjing Jiancheng Bioengineering Institute, Jiangsu, China).

The inflammatory cytokines in serum from rats with repeated TiO_2_ NPs exposure were analyzed by the Cytometric Bead Array (CBA) Rat inflammatory cytokines Flex Set (BD Biosciences, San Jose, CA). Briefly, interleukin 1α (IL-1α), interleukin 4 (IL-4), and tumor necrosis factor (TNF) concentrations were determined using BD FACSCalibur flow cytometers according to the manufacturer’s protocol (BD Biosciences, San Jose, CA). The data were analyzed by FCAP Array Software (Soft Flow Inc., Pecs, Hungary).

### 16S rDNA sequencing and gut microbiota analysis

Genomic DNA of fecal samples was extracted by Cetyltrimethylammonium Ammonium Bromide (CTAB) method after water was removed by freeze-drying apparatus. After the purity and concentration of DNA were detected by agarose gel electrophoresis, DNA samples were diluted with sterile water to 1 ng/μL. PCR amplification was conducted by using the diluted genomic DNA as template, and the specific primers with Barcode, Phusion® High-Fidelity PCR Master Mix with GC Buffer and high-efficiency fidelity enzyme. The PCR amplification system was as follows: 2 × taq PCR mix: 25 μl; Primer F (10 μM): 1 μl; Primer FR (10 μM): 1 μl; gDNA: 2.5 μl; H_2_O: 8.0 μl. The procedure of PCR amplification was as follows: 1) 95 °C for 5 min; 2) Step a-c cycle 34 times, a) 94 °C for 1 min, b) 57 °C for 45 s, c) 72 °C for 1 min; 3) 72 °C for 10 min; 4) 16 °C for 5 min. The primer sequence was as follows (5′-3′): V4-515F, GTGCCAGCMGCCGCGGTAA; V4-806R, GGACTACHVGGGTWTCTAAT; V3 + V4-341F, CCTAYGGGRBGCASCAG; V3 + V4-806R, GGACTACNNGGGTATCTAAT; V4 + V5-515F, GTGCCAGCMGCCGCGGTAA; V4 + V5-907R, CCGTCAATTCCTTTGAGTTT. The V3-V5 region of 16S rRNA gene extracted from fecal specimens was amplified by universal primers. The PCR product was detected by electrophoresis with 2% agarose gel. According to the concentration of PCR product, the sample was mixed equally. After mixing fully, the PCR product was purified by 2% agarose gel electrophoresis with 1 × TAE, and the target band was cut and recycled. GeneJET gel recovery kit was used to recover the purified product. Sequencing libraries were generated using Ion Plus Fragment Library Kit 48 rxns library kit. The libraries were quantified by Qubit fluorescence and then single-End sequencing was performed by Ion S5™XL sequencer. Small fragment libraries were constructed for sequencing. The operation steps in the experiment were strictly in accordance with the instructions.

QIIME (Version 1.9.1) software was used to filter the mosaic data. Subsequently, the sequence data obtained are compared with the sequence in 16S: Gold database, and the chimera sequence is detected and removed to obtain the effective sequence (Clean reads) for subsequent analysis. Uparse (v7.0.1001) software was used to cluster all clean reads and sequences with similarity greater than 97% were clustered into an Operational Taxonomic Unit (OTU). The pseudo-OTUs caused by chimeras were discriminated and filtered. The OTUs of each sample were obtained, and the sequence with the highest frequency of each OTU was selected as the representative sequence. Mothur algorithm and SILVA SSU r132 database were used to annotate the representative sequences of OTUs and obtain taxonomic information. The community compositions of each sample were count at level of kingdom, luphym, class. Order, family, genus and species. Using MUSCLE (Version 3.8.31) software for fast multi-sequence alignment, the phylogenetic relationships of all OTUs representative sequences were obtained. Finally, according to the sequence with the least amount of data in the sample, the data of each sample were normalized. The normalized data was used in the subsequent Alpha and Beta diversity analysis to compare the different bacteria community structure among different experiment groups. KRONA was used to visualize the results of species annotation. The first 10 species with the highest abundance in each taxonomic hierarchy (phylum, class, order, family, genus, species) were selected to draw a cylindrical accumulative map of relative abundance of species generated by taxonomic tree. LEfSe (Linear Discriminant Analysis Effect Size) software was used to compare the species differences among groups. Linear Discriminant Analysis (LDA) was used to find the different intestinal bacteria among groups (LDA Score > 4). In addition, we used Tax4Fun (taxon for function) package in R software to predict the metabolic capacity based on 16S results. The principle is to align the OTUs sequence with the OTUs sequence in SILVA database, and map the OTUs sequence data to the OTUs with macrogenomic information in the corresponding SILVA database by nearest neighbors identification, so as to obtain the predicted macrogenomic data of bacteria. The data were then converted to the abundances of the coding genes of the corresponding enzymes by the prokaryotic KEGG organisms database and NCBI genome annotations. Then, according to the abundances of related enzymes, we can predict the metabolic ability of the gut microbiota to a certain metabolic pathway substance, so as to predict the metabolic pathway changes of the gut microbiota with 16S sequencing data.

### Measurement of lipopolysaccharides (LPS) and short-chain fatty acids (SCFAs)

LPS content in the feces was measured using enzyme-linked immunosorbent assay (ELISA) kits (Wuhan Abebio Science Co., Ltd., China) according to the manufacturer’s instructions. The assay employed a two-site sandwich ELISA to quantitate LPS in samples. SCFAs in the feces, including acetic acid (AA), propionic acid (PA), isobutyric acid (IBA), butyric acid (BA), isovaleric acid (IVA), and hexanoic acid (HA), were assayed by targeted metabonomics using GC-MS/MS (Thermo, USA). The key parameters for GC-MS/MS analysis are shown in Additional file 1: Table S5. The software Agilent Mass Hunter was used for data processing. Quality control (QC) samples were prepared by mixing sample extracts to monitor the repeatability of the analysis process.

### Statistical analysis

The methods of statistical analysis for the metabonomics and gut microbiota data were previously described. Other data were expressed as means ± standard deviations (SDs) and analyzed with SPSS 20.0. One-way analysis of variance (ANOVA) with least significant difference (LSD) or the Dunnet T3 test was applied to evaluate the statistical significance of differences between the experimental groups and the controls. A *p* value < 0.05 was considered to be statistically significant.

## Supplementary information

**Additional file 1: Table S1.** Key parameters for metabolite identification by Compound Discoverer software. **Table S2**. 29 differential metabolites in liver tissues of rats exposed to TiO_2_ NPs orally for 90 days, analyzing by OPLS-DA method. **Table S3**. Pathway analysis of differential metabolites in liver tissues of rats orally exposed to TiO_2_ NPs. **Table S4.** Significant differential metabolic pathways of gut microbiota induced by TiO_2_ NPs, predicting by Tax4Fun. **Table S5.** Key parameters for GC-MS/MS analysis. **Figure S1.**Body weight of the rats after gastrointestinal exposure to TiO_2_ nanoparticles for 90 days (mean ± SD, *n* = 6). **Figure S2.** Food intake of the rats after gastrointestinal exposure to TiO_2_ nanoparticles for 90 days (mean ± SD, *n* = 6). **Figure S3.** Differential metabolites between the liver samples in the control and TiO_2_ NPs (50 mg/kg) treated group. V Score Map of metabolites using OPLS-DA model. **Figure S4.** The structure and composition of gut microbiota communities at class, order, family and genus level (Top ten). **Figure S5.** Detection of elements in liver tissues of rats after oral administration of TiO_2_ NPs for 90 days.

## Data Availability

The datasets used and/or analyzed during the current study are available from the corresponding author on reasonable request.

## Abbreviations

AA: Acetic acid
AGJ: Artificial gastric juice
AIJ: Artificial intestinal juice
ALB: Albumin
ALT: Alanine aminotransferase
AST: Aspartate aminotransferase
BA: Butyric acid
BET: Brunauer–Emmett–Teller
CBA: Cytometric Bead Array
DLS: Dynamic light scattering
EDS: X-ray spectroscopy
GLB: Globulin
GSH: Reduced glutathione
GSH-Px: Glutathione peroxidase
GSSG: Oxidized glutathione
HA: Hexanoic acid
IBA: Isobutyric acid
ICP-MS: Inductively coupled plasma mass spectrometry
IL: Interleukin
IVA: Isovaleric acid
LDA: Linear discriminant analysis
LefSe: Linear discriminant analysis effect size
LPS: Lipopolysaccharides
MDA: Malondialdehyde, lipid peroxidation products
NPs: Nanoparticles
OPLS-DA: Orthogonal projection to latent structure discriminant analysis
PA: Propionic acid
PCA: Principal component analysis
PDI: Poly dispersity index
Qc: Quality control
SCFAs: Short-chain fatty acids
SEM: Scanning Electron Microscopy
SOD: Superoxide dismutase
TBIL: Total bilirubin
TEM: Transmission Electron Microscopy
TiO_2_: Titanium dioxide
TNF: Tumor necrosis factor
TP: Total protein
UPLC-QEMS: Ultra High Performance Liquid Chromatography-Q-Exactive Orbitrap-High-resolution Mass Spectrometry System
XRD: X-ray powder diffractometry
