# Supplementary material for: Hepatotoxicity and the role of the gut-liver axis in rats after oral administration of titanium dioxide nanoparticles
Source: Part Fibre Toxicol. 2019 Dec 27;16:48. doi: 10.1186/s12989-019-0332-2 (PMC6935065; doi:10.1186/s12989-019-0332-2)
Supplement: Supplementary file 1 — Additional file 1: Table S1. Key parameters for metabolite identification by Compound Discoverer software. Table S2. 29 differential metabolites in liver tissues of rats exposed to TiO2 NPs orally for 90 days, analyzing by OPLS-DA method. Table S3. Pathway analysis of differential metabolites in liver tissues of rats orally exposed to TiO2 NPs. Table S4. Significant differential metabolic pathways of gut microbiota induced by TiO2 NPs, predicting by Tax4Fun. Table S5. Key parameters for GC-MS/MS analysis. Figure S1.Body weight of the rats after gastrointestinal exposure to TiO2 nanoparticles for 90 days (mean ± SD, n = 6). Figure S2. Food intake of the rats after gastrointestinal exposure to TiO2 nanoparticles for 90 days (mean ± SD, n = 6). Figure S3. Differential metabolites between the liver samples in the control and TiO2 NPs (50 mg/kg) treated group. V Score Map of metabolites using OPLS-DA model. Figure S4. The structure and composition of gut microbiota communities at class, order, family and genus level (Top ten). Figure S5. Detection of elements in liver tissues of rats after oral administration of TiO2 NPs for 90 days. [file 12989_2019_332_MOESM1_ESM.pdf]

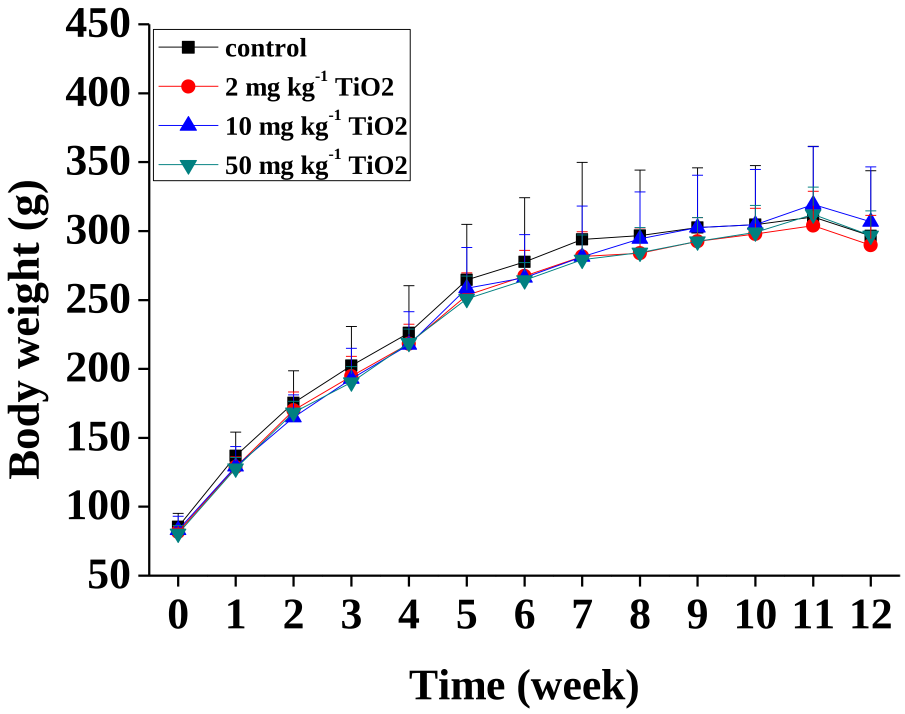

Figure S1. Body weight of the rats after gastrointestinal exposure to TiO_2 nanoparticles for 90 days (mean ± SD, n = 6).

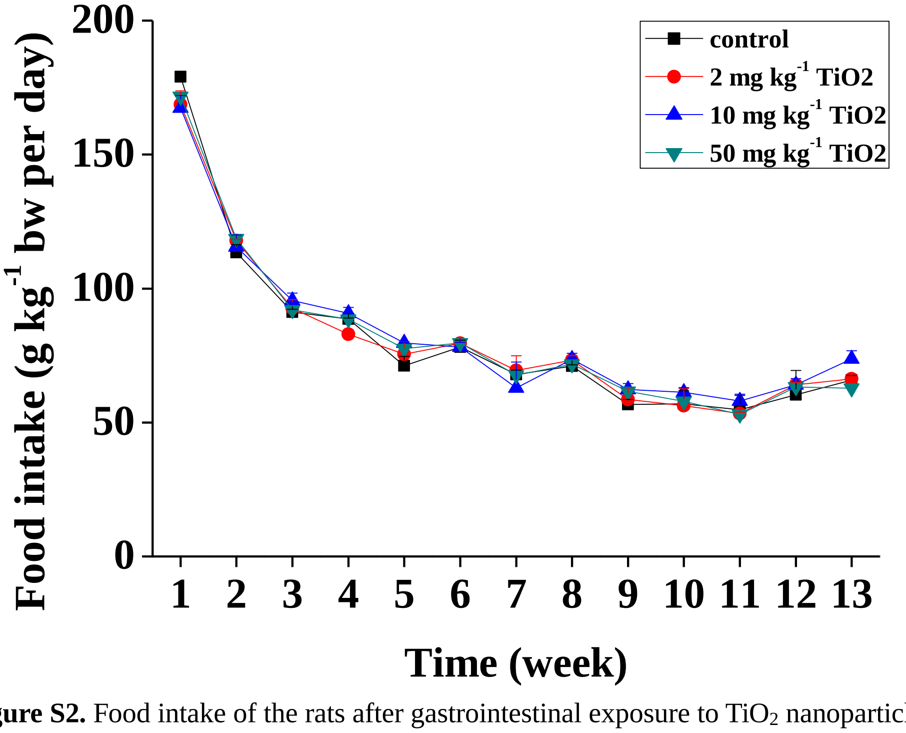

Figure S2. Food intake of the rats after gastrointestinal exposure to TiO_2 nanoparticles for 90 days (mean ± SD, n = 6).

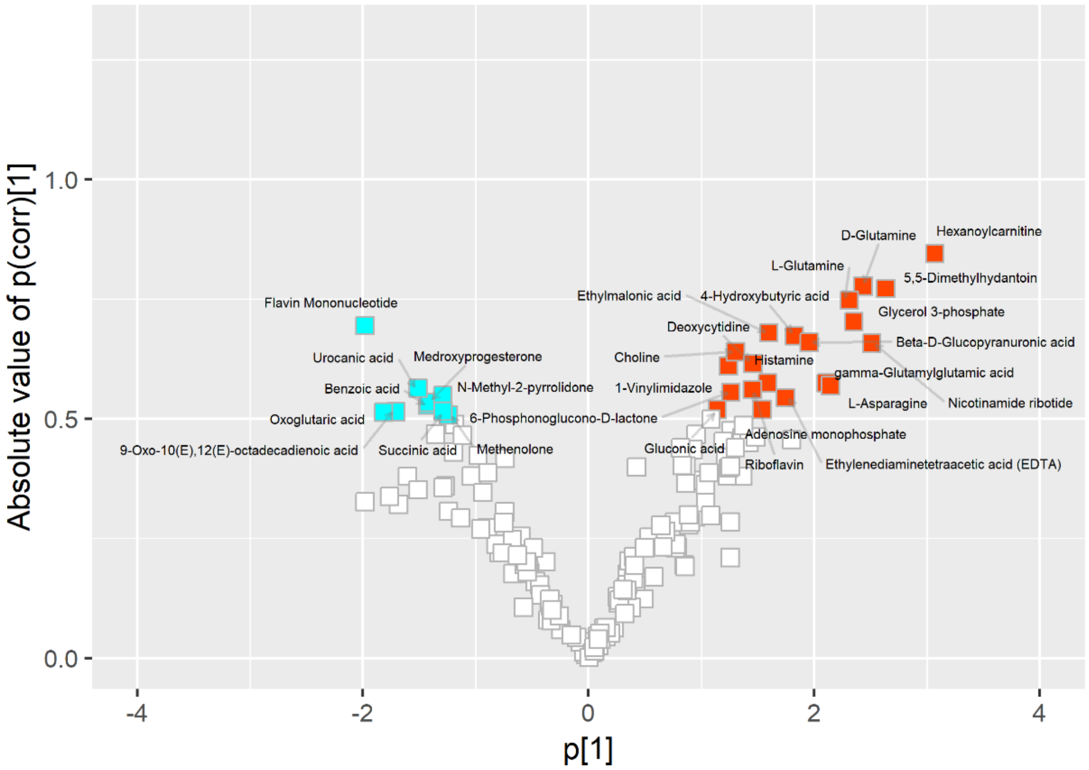

Figure S3. Differential metabolites between the liver samples in the control and TiO_2 NPs (50 mg/kg) treated group. V Score Map of metabolites using OPLS-DA model. X-axis represents the difference between groups and Y-axis represents the reliability of differences between groups. Taking p(corr) > 0.5 as the demarcation value, the red points with name annotations indicate that the concentration of the metabolite in the TiO_2 NPs (50 mg/kg) treated group is higher than that in the control group. The cyan points with name annotations indicate that the concentration of the metabolite in the TiO_2 NPs (50 mg/kg) treated group is lower than that in the control group.

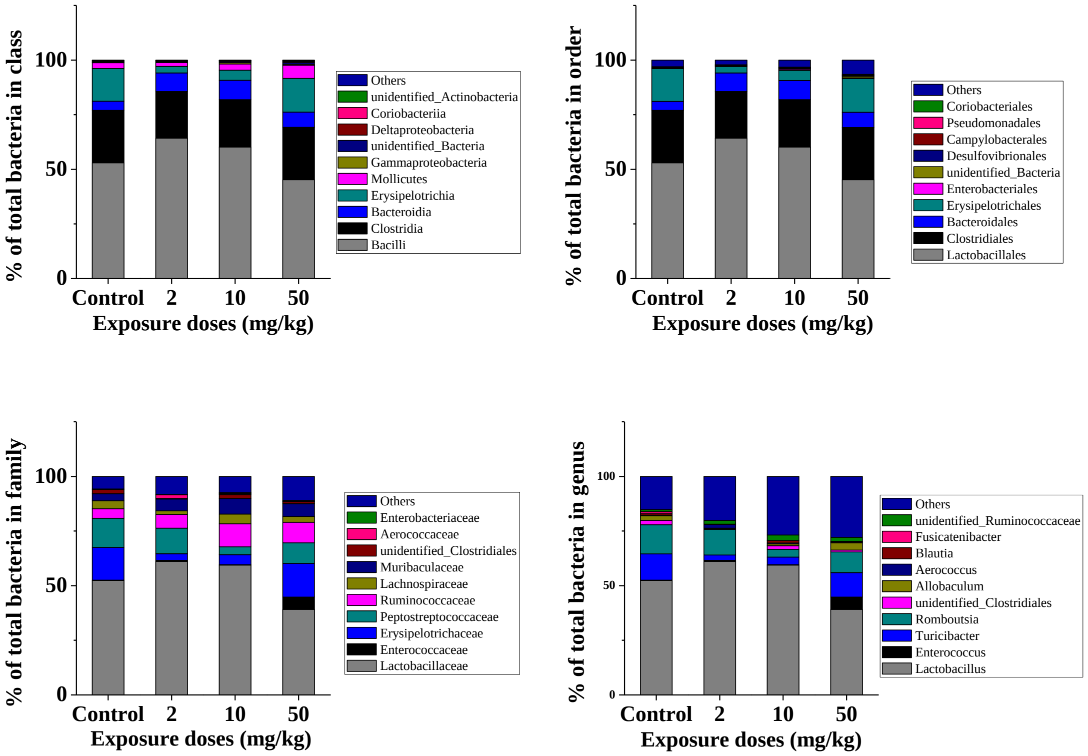

Figure S4. The structure and composition of gut microbiota communities at class, order, family and genus level (Top ten).

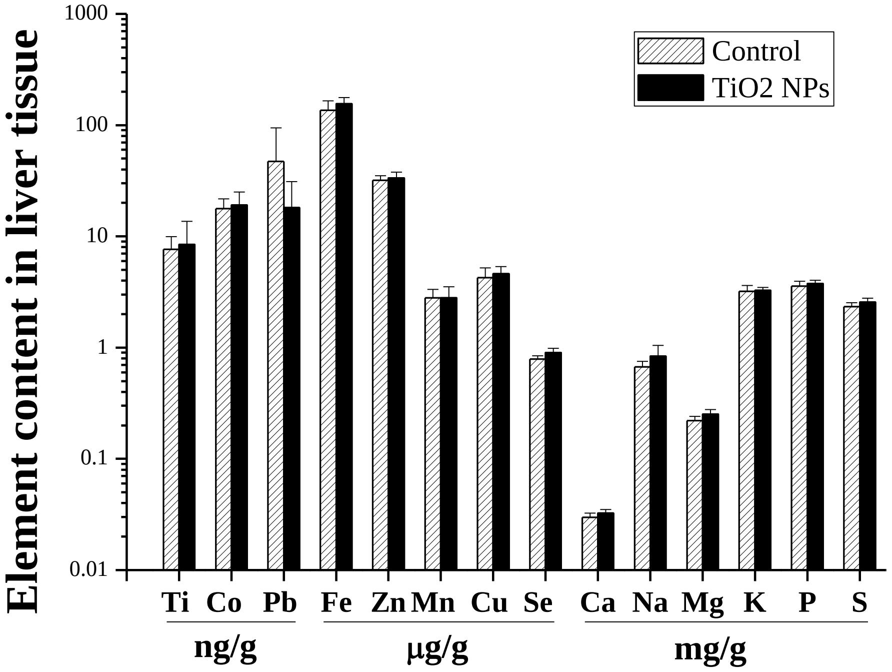

Figure S5. Detection of elements in liver tissues of rats after oral administration of TiO_2 NPs for 90 days.

Table S1. Key parameters for metabolite identification by Compound Discoverer software.

Parameters	QE HF
Alignment// RT tolerance （min）	0.2
Select spectra// min peak count	1
Select spectra// S/N threshold	3
Detect Compounds// S/N threshold	1.5
Alignment// model	Adaptive curve
Min Peak Intensity	500 000
Match Ion Activation Energy	True
Ion Activation Energy Tolerance	50
Fragment Data Selection// Preferred Ions	[M+H]+1; [M-H]-1
Identity Search	HighChem HighRes
Similarity Search	Confidence Forward
Gap filling// S/N thresholda	1.5
Gap filling// Mass Tolerance	5ppm

Table S2. 29 differential metabolites in liver tissues of rats exposed to TiO_2 NPs orally for 90 days, analyzing by OPLS-DA method.

Super class	Metabolite name	Molecular formula	retention time, RT (min)	molecular mass	Maximum peak area	HMDB ID	KEGG ID	Change fold (log_2N)
Benzenoids	Benzoic acid	C7H6O2	4.701	122.037	791251.1	0001870	C00180	-0.53
Lipids and lipid-like molecules	Methenolone	C20H30O2	1.069	302.2231	1662736	-		-0.36
Lipids and lipid-like molecules	Medroxyprogesterone	C22H32O3	1.412	344.2334	869352.6	0001939	C07119	-0.40
Lipids and lipid-like molecules	9-Oxo-10（E）,12（E）-octadecadienoic acid	C18H30O3	1.746	294.218	3155559	-	-	-0.95
Lipids and lipid-like molecules	Hexanoylcarnitine	C13H25NO4	4.1755	259.1771	1088465	0000705	-	1.29
Lipids and lipid-like molecules	4-Hydroxybutyric acid	C4H8O3	7.17	104.0475	1105030	0000710	C00989	0.55
Lipids and lipid-like molecules	Ethylmalonic acid	C5H8O4	8.909	132.0425	621037.9	0000622	-	0.47
Lipids and lipid-like molecules	Glycerol 3-phosphate	C3H9O6P	11.201	172.014	3262750	0000126	C00093	1.02
Nucleosides, nucleotides, and analogues	Deoxycytidine	C9H13N3O4	4.288	227.091	431949.5	0000014	C00881	1.05
Nucleosides, nucleotides, and analogues	Flavin Mononucleotide (FMN)	C17H21N4O9P	10.477	456.1024	194210.8	0001520	C00061	-0.79

Nucleosides, nucleotides, and analogues	Adenosine monophosphate (AMP)	C10H14N5O7P	11.145	347.0636	149332.9	0000045	C00020	0.57
Nucleosides, nucleotides, and analogues	Nicotinamide ribotide (NR)	C11H15N2O8P	12.414	334.0551	2633682	0000229	C00455	-0.41
Organic acids and derivatives	Oxoglutaric acid	C5H6O5	9.205	146.0218	6319472	0000208	C00026	-0.83
Organic acids and derivatives	D-Glutamine	C5H10N2O3	9.252	146.0684	20794166	0003423	C00819	0.50
Organic acids and derivatives	L-Asparagine	C4H8N2O3	9.294	132.0529	797332.7	0000168	C00152	1.14
Organic acids and derivatives	L-Glutamine	C5H10N2O3	9.38525	141.8123	34301818	0000641	C00064	0.93
Organic acids and derivatives	Succinic acid	C4H6O4	9.877	118.0267	36294417	0000254	C00042	-0.45
Organic acids and derivatives	γ-Glutamylglutamic acid	C10H16N2O7	12.025	276.0945	896286.2	0011737	C05282	0.99
Organic acids and derivatives	Ethylenediaminetetraacetic acid （EDTA）	C10H16N2O8	12.135	292.0892	871078.5	-	-	0.54
Organic nitrogen compounds	Choline	C5H13NO	6.794667	103.0995	554000000	0000097	C00114	0.36
Organic nitrogen compounds	Histamine	C5H9N3	7.298	111.0794	3304336	0000870	C00388	0.44

Organic oxygen compounds	Gluconic acid	C6H12O7	9.1145	196.0585	85328050	0000625	C00257	0.36
Organic oxygen compounds	6-Phosphonoglucono-D-lactone	C6H10O6	9.2965	178.048	843505.1	0001127	C01236	0.35
Organic oxygen compounds	β-D-Glucopyranuronic acid	C6H10O7	9.802	194.0429	5905911	0010314	-	0.81
Organoheterocyclic compounds	Riboflavin	C17H20N4O6	4.172	376.139	4223784	0000244	C00255	0.52
Organoheterocyclic compounds	1-Vinylimidazole	C5H6N2	6.259333	94.05309	8946871	-	-	0.31
Organoheterocyclic compounds	Urocanic acid	C6H6N2O2	6.591	138.0428	2745725	0000301	C00785	-0.69
Organoheterocyclic compounds	N-Methyl-2-pyrrolidone	C5H9NO	8.78	99.06842	1291424	-	-	1.37
Organoheterocyclic compounds	5,5-Dimethylhydantoin	C5H8N2O2	9.27	128.0588	629300.9	-	-	1.22

Table S3. Pathway analysis of differential metabolites in liver tissues of rats orally exposed to TiO_2 NPs.

Pathway Name	Match Status	P^a	-log(P)	*Holm* P^b	FDR^c	Impact[d]
D-Glutamine and D-glutamate metabolism	3/5	2.44×10^{-5}	10.6200	0.0019	0.0019	0.6666
Alanine, aspartate and glutamate metabolism	4/24	2.67×10^{-4}	8.2278	0.0213	0.0108	0.2000

[a] Primary P value of pathway enrichment analysis; [b] The P value was corrected by Holm method; [c] The P value was corrected by FDR; [d] The pathway impact was obtained by pathway topology analysis.

Table S4. Significant differential metabolic pathways of gut microbiota induced by TiO_2 NPs, predicting by Tax4Fun.

Metabolic pathways[#]	Group[&]	q25	Median	q75	Mean	SD	p
ko00531 Glycosaminoglycan degradation	1	0.94	0.97	0.99	1.00	0.11	0.0034*
	2	1.22	1.46	1.70	1.50	0.45	
	3	1.42	1.65	1.94	3.55	4.79	
	4	0.98	1.01	1.03	1.01	0.05	
ko04975 Fat digestion and absorption	1	0.53	0.65	1.73	1.00	0.89	0.0159*
	2	0.23	1.20	7.57	5.52	8.47	
	3	5.10	9.96	28.53	71.86	146.11	
	4	0.32	0.80	1.55	1.03	0.98	
ko05322 Systemic lupus erythematosus	1	0.38	0.88	1.35	1.00	0.89	0.0081*
	2	0.51	1.39	5.84	3.06	3.59	
	3	7.73	14.92	25.03	43.66	74.36	
	4	0.19	0.88	1.67	1.02	0.98	

The values shown in the table were standardized statistics based on the average of the control group. # Number and name of metabolic pathway in KEGG database. * Significant difference compared with the control group ($p < 0.05$). [&] Group 1-4: the control group and low, medium, high-dose TiO_2 NPs (2, 10, 50 mg/kg) treated group., respectively.

Table S5. Key parameters for GC-MS/MS analysis.

	Parameters
Injection volume	2 μL
Front Inlet Mode	2:1
Carrier Gas	Helium
Column	DB-FFAP (30m x 0.25mm x 0.25um)
Column Flow	0.85 mL/min
Oven Temperature Ramp	90 ℃ hold on 1min, raised to 130 ℃ at a rate of 15 ℃/min ,raised to 230 ℃ at a rate of 20 ℃/min, hold on 4min, after running for 5 min
Front Injection Temperature	230 ℃
Transfer Line Temperature	230 ℃
Ion Source Temperature	230 ℃
Quad Temperature	150 ℃
Electron Energy	70 eV
Scan mode	MRM
Solvent Delay	3.3 min
